# Medium-chain-length polyhydroxyalkanoates synthesis by *Pseudomonas putida* KT2440 *relA/spoT* mutant: bioprocess characterization and transcriptome analysis

**DOI:** 10.1186/s13568-017-0396-z

**Published:** 2017-05-12

**Authors:** Justyna Mozejko-Ciesielska, Dorota Dabrowska, Agnieszka Szalewska-Palasz, Slawomir Ciesielski

**Affiliations:** 1 Department of Microbiology, Faculty of Biology and Biotechnology, University of Warmia and Mazury in Olsztyn, Oczapowskiego 1A, 10-719 Olsztyn, Poland; 2 Department of Environmental Biotechnology, University of Warmia and Mazury in Olsztyn, Olsztyn, Poland; 3 Department of Molecular Biology, University of Gdansk, Gdansk, Poland

**Keywords:** Global regulation, *Pseudomonas putida* KT2440, Polyhydroxyalkanoates, Stringent response, Transcriptomics

## Abstract

*Pseudomonas putida* KT2440 is a model bacteria used commonly for medium-chain-length polyhydroxyalkanoates (mcl-PHAs) production using various substrates. However, despite many studies conducted on *P. putida* KT2440 strain, the molecular mechanisms of leading to mcl-PHAs synthesis in reaction to environmental stimuli are still not clear. The rearrangement of the metabolism in response to environmental stress could be controlled by stringent response that modulates the transcription of many genes in order to promote survival under nutritional deprivation conditions. Therefore, in this work we investigated the relation between mcl-PHAs synthesis and stringent response. For this study, a *relA*/*spoT* mutant of *P. putida* KT2440, unable to induce the stringent response, was used. Additionally, the transcriptome of this mutant was analyzed using RNA-seq in order to examine rearrangements of the metabolism during cultivation. The results show that the *relA*/*spoT* mutant of *P. putida* KT2440 is able to accumulate mcl-PHAs in both optimal and nitrogen limiting conditions. Nitrogen starvation did not change the efficiency of mcl-PHAs synthesis in this mutant. The transition from exponential growth to stationary phase caused significant upregulation of genes involved in transport system and nitrogen metabolism. Transcriptional regulators, including *rpoS*, *rpoN* and *rpo*D, did not show changes in transcript abundance when entering the stationary phase, suggesting their limited role in mcl-PHAs accumulation during stationary phase.

**Electronic supplementary material:**

The online version of this article (doi:10.1186/s13568-017-0396-z) contains supplementary material, which is available to authorized users.

## Introduction

To survive in the harsh conditions, microorganisms need to be able to adapt to a competitive and changing environment. In response to changes in environmental conditions, the physiological status of microorganisms can change due to the actions of transcriptional regulatory systems. Generally, transcription regulation occurs at two different levels. Firstly, regulation can drive the expression of relevant pathway genes in reaction to a specific inducer. Secondly, global regulatory systems can adjust the expression of the pathway gene cluster in response to the general physiological status of the microorganism. The main regulators at this higher level are alternative RNA polymerase sigma subunits (Díaz and Prieto 2000).

One of the survival strategies, that microorganisms living in stressful environments have developed, is synthesis and accumulation of polyhydroxyalkanoates (PHAs) in aerobic and anaerobic conditions (Poirier et al. 1995). PHAs play an important role in central metabolism because they serve as a reservoir of carbon and reducing equivalents. The most common PHA is poly(3-hydroxybutyrate) (PHB), composed of monomers containing four carbon atoms, whereas medium-chain-length PHAs, containing from 6 to 14 carbon atoms in single monomer, are less abundant. The accumulation of PHAs may increase the survival capabilities of these bacteria in extreme environments or when nutrient availability is poor (Ayub et al. 2009). Because of the complexity of PHAs metabolism knowledge on the re-configuration of the bacteria whole metabolism under the conditions that lead to mcl-PHAs synthesis is still not clear. In *Pseudomonas* species the *pha* cluster is very well conserved and is organized into two operons *phaC1ZC2D* and *phaFI*. Two polymerases (PhaC1 and PhaC2), a depolymerase (PhaZ), a transcriptional activator (PhaD) and proteins involved in granule formations (PhaF and PhaI) are essential to accumulate and synthesize these biopolyesters. It has been suggested that PhaG encodes transacylase, being not co-localized with the *pha* gene cluster, is also involved in mcl-PHAs synthesis from non-related carbon sources (Hoffmann and Rehm 2004). It is known that PHAs are accumulated by pseudomonads mainly in response to unbalanced growth conditions such as a lack of nitrogen, which links PHAs accumulation to the stringent response (López et al. 2015).

The stringent response modifies the physiology of the bacterium to such an extent that it can survive difficult environmental circumstances during its lifecycle. This response is mediated by the alarmons-unusual nucleotides, guanosine tetraphosphate (ppGpp) and guanosine pentaphosphate (pppGpp), often referred collectively to (p)ppGpp, which primarily affects the transcriptional program of the bacterial cell (Potrykus and Cashel 2008). In model bacteria, *Escherichia coli* and many beta- and gammaproteobacteria, including *P. putida*, two enzymes modulate the levels of ppGpp (Mittenhuber 2001; Atkinson et al. 2011): the ppGpp synthetase RelA, responsive to amino acid starvation and ppGpp synthetase/hydrolase SpoT, producing (p)ppGpp under other nutrients limitations and stresses (Potrykus and Cashel 2008). Stringent response contributes to stress adaptation, antibiotic tolerance, expression of virulence traits and acquisition of persistent phenotypes in pathogenic bacteria. The regulation of the mode of action of the RelA/SpoT enzymes has been extensively studied, but the regulatory mechanisms that manage transcription of their genes are still not fully understood (Brown et al. 2014).

Recently, Brigham and co-workers (2012) revealed that the polyhydroxybutyrate production cycle in *Ralstonia eutropha* H16 is regulated by the stringent response. The results indicated that *R. eutropha* mutant unable to produce ppGpp did not accumulate PHB unless the stringent response was chemically induced. Previously, Ruiz et al. (2001) had shown that alarmones accumulation and PHB degradation are associated in *Pseudomonas oleovorans*: as PHB were degraded, ATP and ppGpp levels increased. They suggested that the stringent response influenced PHB utilization by activation of RpoS synthesis. Because of these previous studies and the importance of the stringent response in reprogramming bacterial transcription, we hypothesized that *P. putida* deficient in the stringent response may be impaired in mcl-PHAs synthesis.

Thus, to examine the role of the stringent response in mcl-PHAs synthesis, *P. putida* KT2440, a model organism for mcl-PHAs production, was used. A set of experiments were performed to determine the possibility of mcl-PHAs accumulation by *P. putida* KT2440 mutant deficient in stringent response and to investigate the molecular background of this process. Firstly, in shaking flasks experiments, the mcl-PHAs accumulation bioprocess stimulated by nitrogen starvation in *P. putida* KT2440 *relA*/*spoT* mutant and wild type of *P. putida* KT2440 was compared. Additionally, *P. putida* KT2440 *rpoN* mutant was used in this comparison to examine the role of RpoN in mcl-PHAs synthesis under nitrogen deprivation conditions. Secondly, the cultivation of *P. putida* KT2440 *relA/spoT* mutant in a bioreactor was conducted to monitor cell growth and biopolymers accumulation over time. Moreover, the transcriptome of *P. putida* KT2440 *relA*/*spoT* mutant cultivated in the bioreactor was analyzed in order to show rearrangements of the metabolism during fermentation towards mcl-PHAs synthesis.

## Materials and methods

### Bacterial strain and growth conditions

Cells of *P. putida* KT2440 (ATCC^®^ 47054™), *P. putida* KT2440 *rpoN* mutant (Köhler et al. 1989), and *P. putida* KT2440 *relA*/s*poT* (Sze et al. 2002) mutant were taken from a deep-frozen stock and grown overnight in Luria–Bertani broth (1% w/v tryptone, 0.5% w/v yeast extract, 1% NaCl) with shaking at 30 °C with 200 rpm for 24 h before inoculation. All studied strains were cultivated under nitrogen-limiting and non-limiting conditions. For all cultivations, the nitrogen-limited medium contained the following components per liter: 2 g Na_2_HPO_4_⋅12H_2_O, 14.9 g KCl, 46.72 g NaCl, 14.5 g Tris, 2.05 g MgCl_2_, 3.53 g Na_2_SO_4,_ 1 g (NH_4_)_2_ SO_4_, 1 g MgSO_4_⋅7H_2_O, and 2.5 mL of trace element solution. In the non-limited experiments, the level of (NH_4_)_2_ SO_4_ was adjusted to 10 g/L. Each liter of trace element solution contained per liter: 20 g FeCl_3_⋅6H_2_O, 10 g CaCl_2_⋅H_2_O, 0,03 g CuSO_4_⋅5H_2_O, 0,05 g MnCl_2_⋅4H_2_O, 0,1 g ZnSO_4_⋅7H_2_O dissolved in 0.5 N HCl. All cultures were supplemented with oleic acid (10 mL/L) as the only carbon source in the production media. The 250-mL Erlenmeyer flasks containing 100 mL of a mineral medium were incubated for 48 h at 30 °C in a rotary shaker at 200 rpm. The shaking flasks cultivations were performed in six replicates for each condition and for each strain.

The fermentation study of *P. putida* KT2440 *relA/spoT* mutant was carried out in a 5 L working volume in a bioreactor (BioFlo 110, New Brunswick Scientific) at 30 °C with an aeration rate of 4 L/min. Parameters like dissolved oxygen, pH value, biomass, mcl-PHA, nitrogen and carbon concentrations were controlled during the experiments.

pH-value was maintained at seven through the modulated addition of concentrated 1 N NaOH and 1 N HCl. The dissolved oxygen was monitored during the whole cycle with O_2_ electrode (InPro 6800, Mettler Toledo GmbH, Switzerland). Total fermentation time was 48 h.

### Analytical methods

The samples from shake flasks experiment were taken after 48 h of cultivation in order to measure cell dry weight and PHA concentration. The cell density of the cultures in the bioreactor was monitored by measuring the absorbance at 600 nm (OD600) using a spectrophotometer. During the cultivation in the bioreactor the samples were taken at 8, 17, 24, 32, 41 and 48 h for measurements of cell dry weight, mcl-PHAs accumulation, ammonium/carbon concentration and for determination of monomers composition and their concentrations. To measure cell dry weight (CDW), the cells in 100 mL culture broth were harvested by centrifugation at 11.200×*g* for 10 min, washed twice with hexane to remove unused oleic acid and once with distilled water. The collected cells were then weighed after lyophilization. The lyophilization process was performed by Lyovac GT2 System (SRK Systemtechnik GmbH) for 24 h. Ammonium and total organic carbon (TOC) concentration was measured spectrophotometrically using the Hach Lange DR 2800 spectrophotometer (Hach Lange, Düsseldorf DE) and the LCK303 kit for ammonium and LCK380 kit for TOC according to the manufacturer’s instructions.

Mcl-PHAs were extracted from lyophilized cells using the chloroform/methanol procedure for quantitative and qualitative analysis of biopolymers. The monomeric composition of the purified mcl-PHAs was determined using a methanolysis protocol as described previously (Mozejko and Ciesielski 2014). The concentrations of methyl esters were estimated by a gas chromatography (GC) equipped with a capillary column Varian VF-5 ms with a film thickness of 0.25 μm (Varian, Lake Forest, USA). Pure standards of methyl 3-hydroxy-hexanoate, -octanoate, -nonanoate, -decanoate, -undecanoate, -dodecanoate, -tetradecanoate, -hexadecanoate were used to generate calibration curves for the methanolysis assay. All samples were analyzed in triplicates.

Cell dry weight and PHA concentration analysis in biomass from shake flasks and fermentor were performed in this same way. Student t test was used to find statistically significant differences between biomass and PHA concentration.

### RNA isolation

One aliquot of 20 mL from each of cultures were collected and centrifuged at 4000×*g* to pellet the cells and then transferred to a Falcon tube containing RNAlater solution (Sigma). Total RNA extraction was performed using a commercial RNA extraction kit (A&A Biotechnology) according to the manufacturer’s protocol. Isolated RNA samples were treated with On-Column DNase I Digest Set (Sigma) to remove traces of DNA. Each time the absence of contaminating DNA was proven by PCR reaction. The RNA quantity, quality was checked using capillary electrophoresis (Agilent 2100 Bioanalyzer, California, USA). The RNA integrity number (RIN) of every RNA sample used for sequencing was more than 8.0.

### Reverse transcription PCR analysis

Reverse transcription was performed using a SuperScript Vilo™ cDNA Synthesis Kit (Invitrogen) according to the manufacturer’s instruction. The cDNA reaction for each sample contained 1 μg of total RNA. Samples, without reverse transcriptase (RT) were used as a negative control. The synthesized first strand cDNA was suspended in sterile water and stored at −20 °C. Real-time PCR reaction was performed using SYBR Green technology in an ABI 7500 real-time PCR system (Applied Biosystems, USA) in MicroAmp^TM^ optical 96-well reaction plates (Applied Biosystems, USA). The primer pairs used for real-time amplification are given in Table 1. The reactions were run using the thermal cycling parameters as follows: 95 °C for 3 min, then 40 cycles of 95 °C for 15 s, and 60 °C for 1 min. After performing a run, a final standard melting curve stage was included. In each run, negative controls (no cDNA) for each primer set were included. For quantification of the fluorescence values, a calibration curve was made using dilution series from 5 × 10^−7^ to 5 ng of *P. putida* KT2440 genomic DNA sample. Normalized expression levels of the examined transcripts were estimated relative to the *16S* rRNA gene, as its expression is known to remain relatively constant throughout growth phase of *P. putida*. Then, the concentration of *P. putida* KT2440 DNA was converted to a genome equivalent for calculation of copy numbers in the real-time PCR assays (Cottyn et al. 2011). For the convenience, the genome size of *P. putida* KT2440 (6.18 × 10^6^ bp) available at NCBI (National Center for Biotechnology Information) was used to estimate the mean mass of the *P. putida* KT2440 genome accordingly to the equation:

$${\text{m}} = ({\text{n}} \times {\text{mw}})/{\text{AN}}$$

**Table 1:** Details of the PCR primers used in this study

Name	Amplicon	Sequence	Reference
GlC1 179R	*phaC1*	5′-AAGGTCAACGCCCTGCTGGGT-3′ 5′-GGTGTTGTCGTTGTTCCAGTAGAGGATGTC-3′	(Ciesielski et al. 2010) (Solaiman et al. 2000)
ZRT5 ZRT3	*phaZ*	5′-GAAGTCATCGCCTTTGATGTGCC-3′ 5′-ATCATCCACAGCACCTTGGGCTTG-3′	This study This study
C2RT5 C2RT3	*phaC2*	5′-GCGGCGTGGCTCACCTG-3′ 5′-GAAGCTGTTGGTCGCGCTG-3′	This study This study
D-RTf D-RTr	*phaD*	5′-CATCAGCCCAGGCAACCTGTAC-3′ 5′-GCGCTCGACGATCAAGTGCAG-3′	This study This study
F-RTf F-RTr	*phaF*	5′-GTCATGTTTAGACGGAATACCCAG-3′ 5′-GCGGCCAACCACCAGCTTG-3′	This study This study
I-RTf I-RTr	*phaI*	5′-GCACCGGTCAGCTTCTCGATC-3′ 5′-GGAGCGAACTTGAAGAAGCC-3′	This study This study
phaG2F phaG2R	*phaG*	5′-TTCAAACGCTTCAACTACCGCC-3′ 5′-CGGTCTTGTTCTCCATGTCCAG-3′	This study This study
341F 515R	*16S* rRNA	5′-CCT ACG GGA GGC AGC AG-3′ 5′-AAT CCG CGG CTG GCA-3′	(López-Gutiérrez et al. 2004)

where n is the genome size in base pairs, mw is the average molecular weight per base pairs (660 g/mol), and AN is the Avogadro constant (6.023 × 10^23^ molecules/mol).

### Library construction, illumina sequencing and data analysis

RNAseq template libraries were constructed with 1 μg of the enriched mRNA samples using Truseq RNA Sample Preparation Kit (Illumina, California, USA) according to the manufacturer’s instructions. Deep sequencing was performed by Illumina HiSeq 2500 according to the manufacturer’s description with a read length of 1  ×  50 nucleotides. Sequence reads were pre-processed to trim low-quality reads and filter reads shorter than 20 bp using FASTX Tool Kit. Genome sequences and annotation data of *P. putida* KT2440 were downloaded from NCBI (downloaded on 10 November, 2016). Reads that mapped to non-coding RNA sequences and reads that did not map to unique positions were excluded from further analysis. Remaining reads were mapped to *P. putida* KT2440 genome using Bowtie with the default parameters. The reads per gene values of all genes were calculated from the SAM output files. Testing for differential expression was performed with DESeq and R software package that uses a statistical model based on the negative bionomial distribution (Anders and Huber 2010). Statistical analysis was performed and genes with a false discovery rate (FDR) p value correction <0.05 were determined as differentially regulated genes. The raw RNAseq data were deposited to the NCBI Sequence Read Archive (SRA) database with BioProject accession PRJNA374570.

## Results

### PHAs synthesis in shake flask cultures experiment

**Fig. 1:**
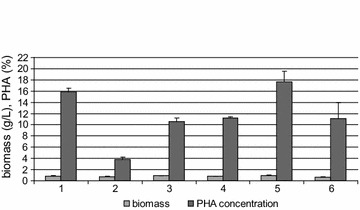
Mcl-PHAs content and biomass concentration of *Pseudomonas putida* KT2440 wild-type (*1* and *2*), *P. putida* KT2440 *relA*/*spoT* mutant (*3* and *4*) and *P. putida* KT2440 *rpoN* mutant (*5* and *6*). The shake flasks cultivation was performed under nitrogen limitation (*1*, *3*, and *5*) and optimal (*2*, *4*, and *6*) conditions. Each data represents the mean ± standard deviation

In order to examine the relationship between the stringent response and mcl-PHAs synthesis, *P. putida* KT2440 and its mutant with non-functional *relA/spoT* genes were cultivated in shaking flasks. Additionally, an RpoN-deficient mutant of *P. putida* KT2440 was used to reveal the role of RpoN in the regulatory network that controls mcl-PHAs synthesis in culture supplemented with oleic acid. All cultivations were carried out in six replicates under optimal growth conditions and under nitrogen limitation. After 48 h of growth, final cell dry weight (CDW) ranged from 0.63 to 0.98 g/L (Fig. 1). Under nitrogen limitation, the wild-form and *rpoN* mutant accumulated the largest amounts of mcl-PHAs (15.9 and 17.7% mcl-PHAs of CDW, respectively). Under optimal conditions, these two strains accumulated significantly less mcl-PHAs (3.8 and 11.1% mcl-PHAs of CDW, respectively; p < 0.05). The *relA*/*spoT* mutant synthesized similar amounts of mcl-PHAs in both optimal and nitrogen limiting conditions (10.6 and 11.2% mcl-PHAs of CDW, respectively). In both conditions the PHA concentration in *relA/spoT* mutant cells was significantly lower than in wild-form cells (p < 0.05).

### PHAs synthesis during bioreactor cultivation

**Fig. 2:**
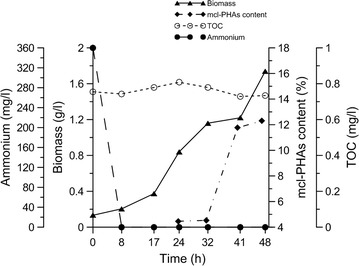
Growth and mcl-PHAs accumulation during 48 h cultivation of *P. putida* KT2440 *relA/spoT* mutant in bioreactor

To characterize the process of mcl-PHAs synthesis by *P. putida* KT2440 *relA*/*spoT* mutant, fed-batch culture was carried out for 48 h in a 5-L bioreactor. The first evidence of mcl-PHAs synthesis was noted at 24 h, but up to 32 h of cultivation, mcl-PHAs concentration remained below 4.5% CDW. After that, mcl-PHAs concentration started to increase rapidly, reaching 12.3% CDW at 48 h, when cell dry weight (1.74 g/L) also reached its maximum value. During fermentation, total nitrogen and phosphorus concentration decreased with time, whereas total carbon concentration was similar throughout cultivation (0.8 g/L). As can be seen in Fig. 2, ammonium was completely consumed during the first 8 h of cultivation

The major repeat units of the mcl-PHAs produced by *P. putida* KT2440 *relA*/*spoT* mutant on oleic acid were 3-hydroxyoctanoate and 3-hydroxyhexanoate, whereas 3-hydroxyhexanoate and 3-hydroxydodecanoate were found in smaller amounts (Table 2). The composition of the PHAs synthesized by this mutant was similar to composition of mcl-PHAs produced by other *Pseudomonas* species cultivated on fatty acids (Ciesielski and Mozejko 2015).

**Table 2:** Monomeric composition of mcl-PHAs synthesized by *Pseudomonas putida* KT2440 *relA/spoT* mutant

Culture time (h)	mcl-PHAs composition (mol%)								
	3HB	3HHx	3HO	3HN	3HD	3HUD	3HDD	3HTD	3HHxD
24	n.d.	n.d.	38.5 ± 5.8	n.d.	41.4 ± 5.4	n.d.	20.1 ± 4.1	n.d.	n.d.
32	n.d.	6.0 ± 1.1	50.0 ± 4.7	n.d.	31.6 ± 5.1	n.d.	12.4 ± 2.5	n.d.	n.d.
41	n.d.	n.d.	45.9 ± 5.5	n.d.	39.4 ± 2.7	n.d.	14.7 ± 1.8	n.d.	n.d.
48	n.d.	6.5 ± 2.0	47.7 ± 11.7	n.d.	32.3 ± 5.9	n.d.	13.5 ± 3.5	n.d.	n.d.

*3HB* 3-hydroxybutyrate, *3HHx* 3-hydroxyhexanoate, *3HO* 3-hydroxyoctanoate, *3HN* 3-hydroxynonanoate, *3HD* 3-hydroxydecanoate, *3HUD* 3-hydroxyundecanoate, *3HDD* 3-hydroxydodecanoate, *3HTD* 3-hydroxytetradecanoate, *3HHxD* 3-hydroxyhexadecanoate, *n.d.* not detected

### Analysis of mcl-PHAs related genes using reverse transcription real-time PCR

**Fig. 3:**
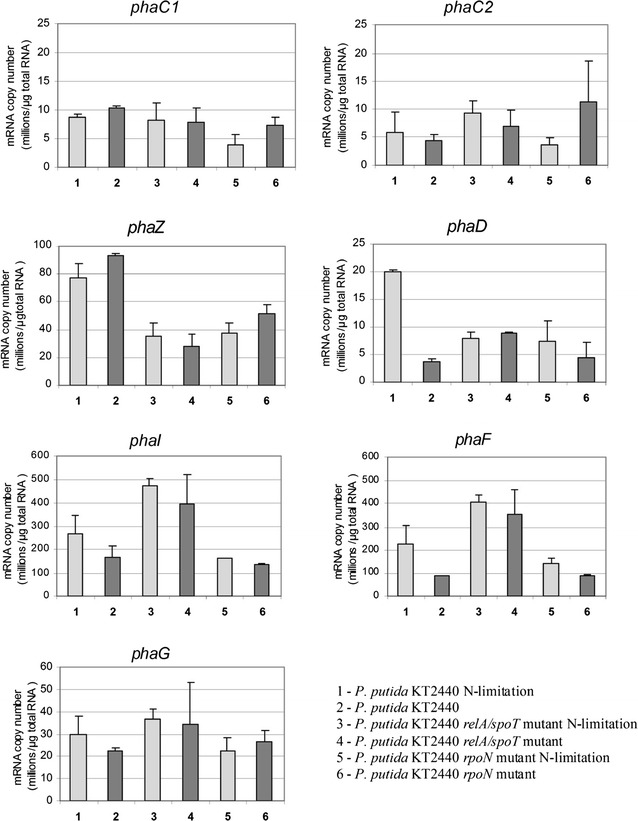
The result of quantitative real-time reverse transcription PCR analysis of *phaC1*, *phaZ*, *phaC2*, *phaD*, *phaI*, *phaF*, and *phaG* genes. Samples were taken at 48 h of cultivation. Each data represents the mean ± standard deviation

The transcriptional expression levels of *phaC1*, *phaZ*, *phaC2*, *phaD*, *phaI*, *phaF*, and *phaG* genes were examined. The transcription of all these genes was investigated in flask cultures at 48 h of cultivation. The results in Fig. 3 show that the mRNA copy numbers varied between analyzed strains and conditions. Nitrogen limiting conditions did not increase transcription of *phaC1*, *phaC2*, and *phaZ* genes in any of the analyzed strains. Under both conditions, expression of the *phaZ* gene was significantly higher in the wild-type than in the mutant. Under nitrogen limiting conditions, the number of *phaD* gene transcripts was significantly higher in the wild-form than in the mutant (20.0 vs. 3.6 million copies). Although *phaD* is considered a possible regulator of the P_C1_ promoter, these results did not show that the changes in *phaD* had any effect on *phaC1*, *phaC2*, and *phaZ* expression.

In all strains, *phaI* and *phaF* expression was much higher than expression of other genes directly involved in PHAs synthesis, and the transcript numbers of *phaI* and *phaF* were higher in nitrogen limiting conditions. Similar profile was observed for *phaG,* although its expression level was rather comparable to *phaZ* gene. Whereas the expression of *phaI*, *phaF*, and *phaG* in the *relA/spoT* mutant was significantly higher than their expression in the other strains, the expression of *phaC1*, *phaC2*, *phaZ*, and *phaD* did not differ significantly between strains.

**Fig. 4:**
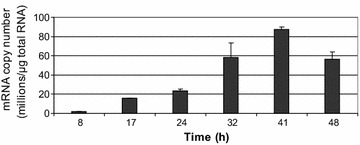
The result of quantitative real-time reverse transcription PCR analysis of *phaG* gene. Samples were taken during cultivation of *P. putida* KT2440 *relA*/*spoT* mutant in bioreactor

Additionally, the expression of *phaG* was investigated at six time-points during *relA*/*spoT* mutant cultivation in the bioreactor (Fig. 4). The *phaG* transcript number increased from the beginning of cultivation until 41 h and then decreased. The changes in *phaG* gene transcription between the exponential growth phase and the stationary phase that were obtained using real-time PCR and RNA-seq were the same.

### Transcriptional analysis using RNA-seq

To investigate how *P. putida* KT2440 *relA/spoT* responds at the molecular level to a decrease in nutrient availability and metabolically adapts to deteriorating conditions, samples were withdrawn for RNA-seq at the end of the exponential phase (24 h) and in the middle of the stationary phase (41 h). The sequence reads matched to 5517 coding genes in the *P. putida* KT2440 genome (Nelson et al. 2002), indicating that the sequencing was deep enough to cover almost all kinds of transcripts in the cells. From the sample withdrawn at 24 h, 18,290,865 sequences of cDNA from mRNA transcripts were obtained; from the sample taken at 41 h, 10,847,701 sequences were obtained.

**Fig. 5:**
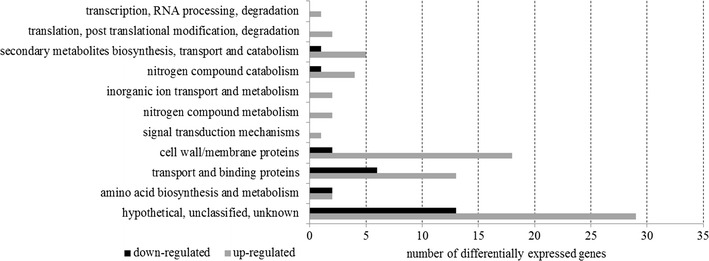
Gene ontology analysis of the significantly differentially expressed genes between exponential growth and stationary phase of *P. putida* KT2440 *relA*/*spoT* mutant cultivation

The RNA-seq analysis revealed that, 104 genes were singnificantly differentially expressed between 24 and 41 h of the cultivation. Most of these differentially expressed genes were up-regulated (78 genes), with fold changes ranging from 8.6 to 106.3 (Additional file 1: Table S1). These genes were classified into categories according to the UniProt annotation pipeline (Fig. 5). With regard to the genes directly involved in mcl-PHAs synthesis or degradation, their expression did not differ significantly between the exponential and the stationary phase.

With the exception of genes coding for proteins involved in amino acid biosynthesis and metabolism, many more genes were upregulated during the stationary phase than were downregulated. Almost half of all the genes showing significant differences in transcription (42 genes) were classified as coding for unknown/hypothetical proteins due to a lack of corresponding genes in databases. The next two largest groups of differentially transcribed genes were associated with cell membrane, cell wall proteins and transport and binding protein. Although there were some downregulated genes in these two groups, as in the group of unknown/hypothetical proteins, in all three groups the number of downregulated genes was less than the number of those that were upregulated. This pattern was also observed in the genes involved in secondary metabolites biosynthesis, transport, and catabolism, and in those responsible for nitrogen compounds catabolism.

More specifically, the genes that were highly upregulated in the stationary phase are mostly involved in the expression of branched-chain amino acid ABC transporters (e.g. *urtA*, *urtC*, *urtD*). Other upregulated genes code for proteins that participate in nitrogen metabolism. Among these genes were the nitrite reductase small (*nirD*) and large (*nirB*) units, and the nitrite transporter (*nasA*). Other upregulated genes code for the urease subunits gamma (*ureA*) and alpha (*ureC*) and for urease accessory proteins (*ureD*, *ureE*, *ureF, ureJ*). Moreover, genes that were upregulated in the stationary phase are involved in fatty acid metabolism: long-chain-fatty-acid-CoA-ligase (PP_2709) and short-chain-dehydrogenase (PP_2711). Furthermore, STRING analysis indicated that some of the significantly upregulated genes that code for hypothetical proteins (PP_2708, PP_2710, PP_2711) are most likely also involved in fatty acids metabolism. Genes involved in the β-oxidation cycle (*fadA, fadB, fadAx, fadBx*) did not show significant changes in their transcription. Most of the downregulated genes belonged to the group of hypothetical proteins, with one exception: transcription of the gene that codes for the amino acid transporter LysE was about 50 times lower in the stationary phase.

Although some genes directly involved in mcl-PHAs synthesis and degradation changed during a shift from exponential growth to stationary phase, these changes were not statistically significant. However, to show even small changes of this genes transcription, the values of RPKM (Reads Per Kilobase per Million) were used to calculate fold-change values (Table 3). Genes coding for *phaC1*, *phaZ*, *phaC2*, *phaF*, and *phaI* showed upregulation in the range from 1.3 to 1.8. *PhaG* gene coding for (R)-3-hydroxydecanoyl-ACP:CoA transacylase showed almost six-fold increase in the stationary phase. Only gene, that showed small downregulation was *phaD*, its calculated fold-change was only at the level of 1.1.

**Table 3:** Differences in *pha* genes transcription between exponential growth (24 h) and stationary phase (41 h) expressed in RPKM

Gene	Locus tag	Description	RPKM 24 h	RPKM 41 h	Fold change	
*phaC1*	PP_5003	PHA polymerase	638.8	834.8	1.3	Up
*phaZ*	PP_5004	PHA depolymerase	128.6	161.2	1.3	UP
*phaC2*	PP_5005	PHA polymerase	164.8	183.2	1.1	UP
*phaD*	PP_5006	Transcriptional regulator	216.6	197.5	1.1	Down
*phaF*	PP_5007	PHA granule-associated	6550.2	9404.0	1.4	Up
*phaI*	PP_5008	PHA granule-associated	3360.2	5976.8	1.8	Up
*phaG*	PP_1408	Acyl-transferase	538.02	3179.0	5.9	Up

Similarly, genes playing the regulatory functions were also investigated accurately (Table 4). All genes coding for RNA polymerase subunits and sigma factors displayed small downregulation. The transcription of Lrp (leucine-responsive regulatory protein) increased about 1.3 in stationary phase, similarly Anr (anaerobic regulatory protein), that showed 1.2 upregulation. Catabolic repression control protein (Crc) changed slightly in the stationary phase (fold-change = 1.1).

**Table 4:** Differences in regulatory genes transcription between exponential growth (24 h) and stationary phase (41 h) expressed in RPKM

Gene	Locus tag	Description	RPKM 24 h	RPKM 41 h	Fold change	
*ntrC*	PP_5048	Fis family transcriptional regulator	62.1	547.7	8.8	Up
*glnK*	PP_5234	Regulatory protein	8152.0	47668.9	5.8	Up
*lrp*	PP_5271	Leucine-responsive regulatory protein	396.2	499.0	1.3	Up
*psrA*	PP_2144	Transcriptional repressor, TetR family	2028.2	1349.0	1.5	Down
*rpoS*	PP_1623	Sigma factor RpoS	9969.7	9452.2	1.1	Down
*rpoD*	PP_0387	Sigma factor RpoD	2845.4	2825.1	1.1	Down
*rpoN*	PP_0952	Sigma factor RpoN	699.2	558.1	1.3	Down
*rpoA*	PP_0479	RNA polymerase subunit alpha	5534.4	4088.8	1.4	Down
*rpoB*	PP_0447	RNA polymerase subunit beta	1443.0	1112.9	1.3	Down
*rpoC*	PP_0448	RNA polymerase subunit beta	1716.1	1329.8	1.3	Down
*crc*	PP_5292	Catabolite repression control protein	1860.3	1655.0	1.1	Down
*gacS*	PP_1650	Sensor kinase	68.7	97.9	1.4	Up
*gacA*	PP_4099	DNA-binding response regulator GacA	1413.5	1169.3	1.2	Down
Unknown gene	PP_2475	Transcriptional regulator, TetR family	1296.3	17129.2	13.2	Up
Unknown gene	PP_1863	Transcriptional regulator, LysR family	154.7	307.5	1.9	Up

Most of the transcriptional regulators showed only small changes in expression between exponential growth and stationary phase (Table 4). The first exception was transcriptional regulator from Fis family (*ntrC*), the main nitrogen stress factor, that showed almost ninefold upregulation. The second exception was transcriptional regulator from TetR family that was activated in stationary phase (fold change 13.2).

## Discussion

The stringent response is a global regulatory system, which mediates major changes in gene expression in response to growth-limiting stress conditions. Because, polyhydroxyalkanoates accumulation is a central feature of survival physiology when cells are stressed, it was hypothesized that these two mechanisms are linked. A set of cultivations performed both in flask cultures and in a bioreactor showed that a *P. putida* KT2400 mutant with non functional *relA*/*spoT* genes is able to produce and accumulate mcl-PHAs. The result of this examination is surprising in the light of observations made by Brigham et al. (2012), who showed that poly(3-hydroxybutyrate) (PHB) is regulated by the stringent response in *R. eutropha* H16. In their study, a *R. eutropha spoT2* mutant accumulated no detectable PHB under conditions of nitrogen starvation, confirming the hypothesis that guanosine tetraphosphate (ppGpp) plays an important role in the production of PHB. A possible relationship between the stringent response and PHB utilization was shown previously by Ruiz et al. (2001) in *P. oleovorans* GPo1. ATP and ppGpp levels increase concomitantly with PHAs degradation in *P. oleovorans* cells. It was postulated that ppGpp is an activator of RpoS synthesis that controls the genes involved in PHB metabolism (Ruiz et al. 2001; Brigham et al. 2012). Here, we show that nitrogen limitation positively influences mcl-PHAs synthesis both in the wild strain and the *rpoN* mutant of *P. putida*, but in RpoN-independent manner as it was shown by Hoffmann and Rehm (2004). Although this observation suggests that this process is dependent mainly on nitrogen availability, nitrogen limitation did not change the efficiency of mcl-PHAs synthesis in the *relA*/*spoT* mutant. Under the stress conditions, in the wild type cells, elevated amount of ppGpp would destabilize RNA polymerase complexes with housekeeping sigma factor promoting transcription from stress related promoters that depend on alternative sigma factors with lower affinity to core RNA polymerase. In this situation, as a result of ppGpp-deficiency due to the mutations, the transcription from the stress-responsive promoters can be impaired, partially due to the insufficient pool of free RNA polymerase core molecules that could bind to other σ factors related to stress tolerance, such as RpoN or RpoS (Potrykus and Cashel 2008).

In the flask culture experiment, the transcription of the genes directly involved in mcl-PHAs synthesis and degradation differed depending on the strain of bacteria and on the availability of nitrogen. The transcription of *phaC1* and *phaC2* were similar in all strains in both conditions. This observation is in contrary to results of Hoffmann and Rehm (2005) who showed that *phaC1* gene expression was slightly induced in *P. putida* KT2440 under nitrogen starvation when sodium gluconate was used. Transcription of *phaZ* was significantly upregulated in *P. putida* KT2440 wild-type under both optimal and nitrogen limiting conditions. It could be suggested that both mutants do not effectively activate processes leading to recovery of energy from PHAs. The number of transcripts of *phaC1, phaZ, phaC2* and *phaD* differed significantly from those of *phaF* and *phaI*, which indicates that these operons are differentially regulated, as in de Eugenio et al. (2010). According to previous studies (Klinke et al. 2000; de Eugenio et al. 2010), the *phaD* gene acts as an activator of the *phaC1* and *phaI* promoters. In our study, however, *phaC1* was not induced by high expression of *phaD* gene in wild type of *P. putida* KT2440 under nitrogen limiting conditions. On the contrary, obtained results support the possible regulation of the *phaI* promoter by *phaD*, which controls the *phaI* and *phaF* gene (Prieto et al. 1999). This was reflected in the higher expression of the *phaI* and *phaF* genes by this strain under nitrogen limitation than under optimal conditions. Because the expression of *phaD* gene was highest in the wild type strain cultivated under nitrogen limiting conditions, it could be suggested that this gene induction is dependent on nitrogen availability. In *rpoN* mutant the difference in *phaD* gene expression was smaller between conditions, therefore *phaI* and *phaF* genes expression difference between conditions was also smaller. Because the expression level of *phaD* gene was much lower in *rpoN* mutant than in wild type under nitrogen limiting conditions it could be speculated that a regulation of this gene could be RpoN dependent. Accordingly to Hoffmann and Rehm (2005), RpoN might be a negative regulator of *phaF* transcription, particularly when excess nitrogen is available, however it was not observed in our study because the expression of *phaF* gene was at the same level both in wild type and *rpoN* mutant. In a case of *relA*/*spoT* mutant, *phaD* gene expression was independent on the used conditions. It is worth emphasizing that, in the *relA*/*spoT* mutant, the *phaI*/*phaF* genes expression was at comparable level in both conditions. Expression of these genes in *relA*/*spoT* mutant was significantly higher than in wild-type strain and *rpoN* mutant, which could suggest that this operon is regulated by the stringent response in negative manner.

RNA-seq analysis revealed that genes directly involved in mcl-PHAs synthesis and degradation in *relA*/*spoT* mutant during cultivation in bioreactor did not show statistically significant changes in transcription between exponential growth and stationary phase. Similarly to the results obtained by Poblete-Castro et al. (2012) and Fu et al. (2015), the highest upregulation was noticed for *phaI* and *phaF* genes. In the mentioned reports, as well as in this study, upregulation of *phaI* was higher, that confirm superiority of *phaI* in relation to *phaF* gene (de Eugenio et al. 2010). The higher induction of *phaI*/*phaF* operon in comparison to *phaC1*/*phaZ*/*phaC2*/*phaD* operon, as well as its significantly higher transcription in flasks culture confirms both independent regulation of *phaI*/*phaF* genes, and possible negative influence of stringent response on these genes expression. The highest upregulation (5.9 fold-change) showed *phaG* gene linking fatty acid *de novo* biosynthesis with PHAs biosynthesis when non-related carbon sources are utilized (Hoffmann and Rehm 2004). The flasks culture showed that its transcription was nitrogen dependent but RpoN-independent. The same observation was previously made by Hoffman and Rehm (2004), when sodium gluconate was used as a carbon source. However, in this work oleic acid was used as the only carbon source, therefore high induction of this gene is surprising. In order to prove this observation *phaG* gene transcription was examined at six time-points during *relA*/*spoT* mutant cultivation in bioreactor using reverse transcription real-time PCR. Obtained results showed gradual increase of this gene transcription until 41 h of cultivation, confirming the results from RNA-seq. It seems that this gene must have (an) additional function(s) in *P. putida* KT2440 metabolism.

Global transcriptomics of the *relA*/*spoT* mutant revealed that only some regulatory genes showed upregulation although these changes were not statistically significant. During the stationary phase the highest activation was shown by genes coding for NtrC transcriptional regulator (fold-change 8.8) and an undetermined gene coding for a transcriptional regulator from TetR family (fold-change 13.2). NtrC, a global regulator, activates the transcription of many genes for the uptake and catabolism of various nitrogen sources and should be activated only during nitrogen limitation (Chubukov et al. 2014). NtrC activated *relA* gene during nitrogen starvation, therefore it is accepted that NtrC couples nitrogen starvation stress and stringent response (Brown et al. 2014). One of the other genes regulated by NtrC is *glnK*. The PII-like protein that is produced by this gene regulates multiple cellular functions related to nitrogen metabolism, including ammonium transport and assimilation via glutamine synthetase, nitrogen fixation and nitrogen-responsive transcriptional regulation, by means of protein–protein interactions (García-González et al. 2009). Our results showed its upregulation, which is in accordance with the results of Poblete-Castro et al. (2012). In their work *glnK* showed a large change in both mRNA abundance and protein level when *P. putida* was subjected to dual limitation of nitrogen and carbon.

One of the regulators specific to the stationary phase is highly conserved bacterial protein Lrp (leucine-responsive regulatory protein), which can act both as a transcriptional repressor and activator (Pletnev et al. 2015) influencing on more than 400 genes in *E. coli*. Among them there are genes responsible for amino acid synthesis, catabolism and the utilization of various carbon sources (Tani et al. 2002). Most probably, Lrp is upregulated by ppGpp in *P. putida* KT2440 like in *E. coli*, which would explain the fact that the increase in abundance of Lrp transcripts during the transition to the stationary phase was not significant.

The RpoS factor, encoded by *rpoS*, activates the transcription of genes involved in the bacterial general stress response. Therefore, it was expected that the number of *rpoS* gene transcripts will increase entering stationary phase. However, the number of *rpoS* gene transcripts did not increase in the *relA/spoT* mutant; the same was observed with the *rpoN* and *rpoD* genes. The observed relation between *rpoS* expression and the stringent response is in agreement with previous reports. For instance, high ppGpp levels resulted in increased *rpoS* transcription in *E. coli* (Gentry et al. 1993), and the *P. aeruginosa relA* mutant strain, which synthesizes less ppGpp than the wild type, had reduced but not abolished RpoS protein levels (Erickson et al. 2004). In *E. coli*, poor induction of the RpoS regulon in the ppGpp-null mutant likely results from lower induction of the *rpoS* gene, weak stabilization of RpoS by IraP, and poor competition of core RNAP for RpoS (Traxler et al. 2008).

The Crc (catabolite repression control) protein is a key regulator involved in the repression by catabolites in *Pseudomonas* at translational level. Generally, the action of Crc is limited to the exponential growth phase and it is not observed when the cultures entered into the stationary phase (La Rosa et al. 2014). In our study the number of mRNA transcripts of Crc decreased in the stationary phase, but they were still present, most likely reducing mcl-PHAs synthesis by repressing *phaC1* translation.

The gene whose expression was upregulated the most was a transcriptional regulator from the TetR family (PP_2475) (fold change 13.2). Follonier et al. (2013) found that when *P. putida* KT2440 was grown in medium supplemented with octanoate, the same gene was upregulated to a much lesser extent under elevated total pressure and elevated oxygen pressure (fold change of 1.59 and 1.7, respectively). A similar upregulation (1.56 fold-change) of a TetR family transcriptional regulator was shown by Fu et al. (2015) for *P. putida* LS46 grown on waste fatty acid but not on glycerol. The authors postulated that this factor is responsible for short chain fatty acid degradation.

In this study 104 genes were significantly differentially expressed between exponential growth and stationary phase. Most of them were responsible for the expression of the branched-chain amino acid ABC transporters and proteins engaged in nitrogen metabolism. Similar observation was made by Poblete-Castro et al. (2012) when wild-type of *P. putida* KT2440 was grown under nitrogen-limitation. Authors observed that expression of the branched-chain amino acid ABC transporter was up to 16-fold higher as a result of stress. Other upregulated genes were responsible for the urea assimilation system (e.g. UreE, UreJ, and UreA). The activation of urease may increase competitive fitness of bacteria under nitrogen-limiting conditions, since urease catalyzes the hydrolysis of urea to yield ammonia. According to Poblete-Castro et al. (2012) the same system was activated both under nitrogen limitation and under carbon–nitrogen dual limitation. However, our examination showed that upregulation of the genes responsible for branched-chain amino acid ABC transporters and nitrogen metabolism was much stronger than in the above mentioned paper. The transcription of genes coding for branched-chain amino acid ABC transporters was even more than 100 times higher in the stationary phase (*utrA*). Similarly, nitrogen metabolism related genes showed more than 100 times higher activity in the stationary phase (e.g. *nirD*). The differences in gene transcription between exponential growth and the stationary phase in the *relA*/*spoT* mutant were unexpectedly high in comparison to values obtained by other authors for *P. putida* KT2440 (Poblete-Castro et al. 2012; Follonier et al. 2013). It is postulated that most of the highly upregulated genes are stringent response dependent. This group comprises genes coding for branched-chain amino acid ABC transporters and nitrogen metabolism. Especially, *glnK* gene coding for nitrogen regulatory protein P-II showed unusually high number of transcripts in the stationary phase (RPKM = 47668.9). This hypothesis could be supported by results obtained for *Corynebacterium glutamicum* (Brockmann-Gretza and Kalinowski 2006). In this study, the *C. glutamicum rel* mutant was used to reveal genes controlled by the stringent response. According to the results obtained by Brockmann-Gretza and Kalinowski (2006), many genes coding for branched-chain amino acid ABC transporters and nitrogen metabolism were negatively controlled by the stringent response. High expression of *phaI* and *phaF* genes observed during the experiment in flasks suggests that these PHA granule associated proteins can be also negatively regulated by ppGpp. Other genes are positively affected by the stringent response. Among them are genes involved in global regulation, with *rpoS* being the most important (Brockmann-Gretza and Kalinowski 2006). RpoS controls the general stress response therefore it plays an important role in adaptation to nutritional and environmental stress. In contrary to *P. putida* deficient in stringent response examined in this study, RpoS expression in the wild type strain is increasing during transition from exponential growth to stationary phase when ppGpp are at the sufficient level. Similar behaviour was observed in the case of genes encoding Lpr, PsrA and GacA transcriptional regulators, that are likely positively affected by stringent response. It is known that *psrA* and *gacA* are involved in *rpoS* activation (Venturi 2003), thus RpoS is additionally restricted. The stringent response deficiency makes cellular metabolism disordered by transcriptional regulators deactivation, and therefore cells are much more unbalanced in a stressful environment.

In conclusion, we showed that the *P. putida* KT2440 *relA*/*spoT* mutant is able to accumulate mcl-PHAs, therefore it could be postulated that the stringent response is not necessary for polyhydroxyalkanoates synthesis using oleic acid as an external carbon source. The composition of mcl-PHAs monomers was similar to that of mcl-PHAs synthesized by other *Pseudomonas* species using fatty acids. Comparative analysis showed that nitrogen limitation did not influence mcl-PHAs synthesis, suggesting that the global regulator did not act properly in the studied mutant. Expression of *phaI*/*phaF* genes in the *relA*/*spoT* mutant was significantly higher than in the wild type strain and the *rpoN* mutant, which suggests that this operon is negatively regulated by the stringent response. The small changes in mcl-PHAs related genes between the exponential growth and stationary phases can be due to the fact that they are activated more or less directly by the RpoS global regulator. Surprisingly, the substantial activation of the *phaG* gene was observed. RNA-seq analysis showed that the transition from exponential growth to the stationary phase caused significant changes in genes encoding the branched-chain amino acid ABC transporters and proteins engaged in nitrogen metabolism. Most of these genes were upregulated, confirming that the stringent response affected their transcription negatively in *P. putida* KT2440. Transcriptional regulators, including *rpoS*, *rpoN* and *rpoD*, did not show changes when entering the stationary phase, therefore it could be suggested that they are influenced positively by stringent response. Although global regulation was non-functioning in the *P. putida* KT2440 *relA*/*spoT* mutant, mcl-PHAs synthesis was made possible by the activity of other transcriptional factors that remain to be determined.

## Additional file

**Additional file 1: TableS1.** Significantly differentially expressed genes in the stationary phase (41 h) with adjusted p-value (Adj. p-value) lower than 0.05. The genes are sorted according to fold-change.

## Abbreviations

PHA: polyhydroxyalkanoates
mcl-PHA: medium-chain-length polyhydroxyalkanoates
PHB: poly(3-hydroxybutyrate)
*P. putida*: *Pseudomonas putida*
ppGpp: guanosine tetraphosphate
(p)ppGpp: guanosine pentaphosphate
ATP: adenosine triphosphate
CDW: cell dry weight
GC: gas chromatography
TOC: total organic carbon
RIN: RNA integrity number
RT: reverse transcriptase
NCBI: National Center for Biotechnology Information
FDR: false discovery rate
RPKM: Reads Per Kilobase per Million
3HB: 3-hydroxybutyrate
3HHx: 3-hydroxyhexanoate
3HO: 3-hydroxyoctanoate
3HN: 3-hydroxynonanoate
3HD: 3-hydroxydecanoate
3HUD: 3-hydroxyundecanoate
3HDD: 3-hydroxydodecanoate
3HTD: 3-hydroxytetradecanoate
3HHxD: 3-hydroxyhexadecanoate
n.d.: not detected
*C. glutamicum*: *Corynebacterium glutamicum*
