# Supplementary material for: Medium-chain-length polyhydroxyalkanoates synthesis by Pseudomonas putida KT2440 relA/spoT mutant: bioprocess characterization and transcriptome analysis
Source: AMB Express. 2017 May 12;7:92. doi: 10.1186/s13568-017-0396-z (PMC5427061; doi:10.1186/s13568-017-0396-z)
Supplement: Supplementary file 1 — Additional file 1: TableS1. Significantly differentially expressed genes in the stationary phase (41 h) with adjusted p-value (Adj. p-value) lower than 0.05. The genes are sorted according to fold-change. [file 13568_2017_396_MOESM1_ESM.docx]

**SUPPLEMENTARY MATERIAL**

AMB Express

**Medium-chain-length polyhydroxyalkanoates synthesis by *Pseudomonas putida* KT2440 *relA/spoT* mutant – bioprocess characterization and transcriptome analysis**

**Justyna MOZEJKO-CIESIELSKA^1*^, Dorota DABROWSKA^2^, Agnieszka SZALEWSKA-PALASZ^3^, Slawomir CIESIELSKI^4^**

^1^ Department of Microbiology, University of Warmia and Mazury in Olsztyn, Poland, e-mail: justyna.mozejko@uwm.edu.pl

^2^ Department of Environmental Biotechnology, University of Warmia and Mazury in Olsztyn, Poland,
e-mail: [dorota.dabrowska@uwm.edu.pl](mailto:dorota.dabrowska@uwm.edu.pl)

^3^ Department of Molecular Biology, University of Gdansk, Poland, e-mail: [agnieszka*.*szalewska*-*palasz*@*biol.ug.edu.pl](mailto:agnieszka.szalewska-palasz@biol.ug.edu.pl)

^4^Department of Environmental Biotechnology, University of Warmia and Mazury in Olsztyn, Poland,
e-mail: slawomir.ciesielski@uwm.edu.pl

**^*^** Corresponding author:

Justyna Mozejko-Ciesielska, Department of Microbiology, Faculty of Biology and Biotechnology, University of Warmia and Mazury in Olsztyn, Oczapowskiego 1A, 10-719 Olsztyn, e-mail: justyna.mozejko@uwm.edu.pl, phone: (+48) (89) 5234234

**Table S1.** **Significantly differentially expressed genes in the stationary phase (41 h) with adjusted p-value (Adj. p-value) lower than 0.05. The genes are sorted according to fold-change**

| Gene_id | Gene name | Adj. p-value | fold-change | |
| --- | --- | --- | --- | --- |
| PP_4841[urtA] | branched-chain amino acid ABC transporter substrate-binding protein | 0.001873198 | 106.33 | up |
| PP_1706[nirD] | nitrite reductase (NAD(P)H) small subunit | 3.65E-05 | 100.09 | up |
| PP_2686[no_symbol] | transglutaminase | 0.001879145 | 81.95 | up |
| PP_3675[no_symbol] | cytochrome c-type protein | 0.006162443 | 71.34 | up |
| PP_2389[no_symbol] | hypothetical protein | 0.004879453 | 62.24 | down |
| PP_1400[kgtP] | metabolite/H+ symportermajor facilitator superfamily metabolite/H+ symporter | 0.003188156 | 61.32 | up |
| PP_1705[nirB] | nitrite reductase (NAD(P)H) large subunit | 0.001724999 | 57.96 | up |
| PP_2688[no_symbol] | hypothetical protein | 0.003864062 | 57.22 | up |
| PP_1742[yjcH] | hypothetical protein | 0.011598709 | 51.59 | down |
| PP_4844[urtD] | Branched-chain amino acid ABC transporter  ATP-binding protein | 0.001873198 | 51.11 | up |
| PP_2388[no_symbol] | amino acid transporter LysE | 0.001873198 | 50.94 | down |
| PP_2092[nasA] | nitrite transporter | 0.000604085 | 48.34 | up |
| PP_4348[no_symbol] | cystathionine beta-lyase | 0.001873198 | 48.22 | up |
| PP_2093[nasT] | response regulator receiver and ANTAR  domain-containing protein | 0.000874094 | 47.84 | up |
| PP_4842[no_symbol] | branched-chain amino acid ABC transporter permease | 0.000604085 | 46.30 | up |
| PP_3618[no_symbol] | hypothetical protein | 0.003864062 | 45.01 | up |
| PP_3470[no_symbol] | hypothetical protein | 0.001873198 | 41.44 | up |
| PP_4087[no_symbol] | hypothetical protein | 0.003006241 | 38.95 | up |
| PP_4293[no_symbol] | hypothetical protein | 0.007874682 | 38.10 | up |
| PP_4631[no_symbol] | hypothetical protein | 0.007219487 | 37.22 | up |
| PP_2687[no_symbol] | hypothetical protein | 0.003864062 | 36.44 | up |
| PP_4578[no_symbol] | major facilitator family transporter | 0.005291939 | 36.21 | up |
| PP_4409[no_symbol] | phage integrase site specific recombinase | 0.00443678 | 35.91 | up |
| PP_5172[no_symbol] | hypothetical protein | 0.01179511 | 32.95 | down |
| PP_3113[no_symbol] | ISPpu13. transposase Orf1 | 0.019262155 | 32.76 | up |
| PP_3468[no_symbol] | hypothetical protein | 0.003864062 | 31.76 | up |
| PP_4637[no_symbol] | 5-methyltetrahydropteroyltriglutamate /homocysteine S-methyltransferase | 0.028530446 | 31.12 | down |
| PP_0236[ssuE] | NAD(P)H-dependent FMN reductase | 0.014506693 | 29.81 | down |
| PP_5171[cysP] | sulfate ABC transporter substrate-binding protein | 0.043608716 | 29.18 | down |
| PP_4843[urtC] | branched-chain amino acid ABC transporter permease | 0.003864062 | 29.06 | up |
| PP_2387[no_symbol] | hypothetical protein | 0.021891514 | 28.62 | down |
| PP_3783[syrB] | hypothetical protein | 0.032261282 | 28.10 | down |
| PP_2681[no_symbol] | pyrroloquinoline quinone biosynthesis protein PqqD | 0.005291939 | 24.98 | down |
| PP_2842[ureD] | urease accessory protein UreD | 0.003864062 | 24.47 | up |
| PP_4297[gcl] | glyoxylate carboligase | 0.022173858 | 24.38 | up |
| PP_4433[no_symbol] | amino acid MFS transporter | 0.007946684 | 24.10 | up |
| PP_2666[no_symbol] | hypothetical protein | 0.007897979 | 23.94 | down |
| PP_3617[no_symbol] | hypothetical protein | 0.012890551 | 23.86 | up |
| PP_2844[ureB] | urease subunit beta | 0.002192846 | 23.37 | up |
| PP_3616[no_symbol] | hypothetical protein | 0.025293312 | 23.22 | up |
| PP_4349[no_symbol] | hypothetical protein | 0.024076683 | 23.06 | up |
| PP_4095[no_symbol] | hypothetical protein | 0.002579274 | 22.50 | up |
| PP_1864[no_symbol] | hypothetical protein | 0.012364137 | 22.02 | down |
| PP_0233[tauA] | taurine ABC transporter substrate-binding  protein | 0.018082879 | 21.39 | down |
| PP_3326[no_symbol] | hypothetical protein | 0.011799103 | 21.28 | down |
| PP_2440[ahpF] | alkyl hydroperoxide reductase | 0.024498454 | 21.20 | up |
| PP_4086[no_symbol] | hypothetical protein | 0.003842161 | 20.91 | up |
| PP_4347[no_symbol] | hypothetical protein | 0.007897979 | 20.45 | up |
| PP_5189[no_symbol] | hypothetical protein | 0.003864062 | 19.72 | up |
| PP_5137[fbpC] | ABC transporter ATP-binding protein | 0.003864062 | 19.55 | up |
| PP_2843[ureA] | urease subunit gamma | 0.005291939 | 19.25 | up |
| PP_2848[ureF] | urease accessory protein UreF | 0.005291939 | 18.71 | up |
| PP_2209[phnW] | 2-aminoethylphosphonate--pyruvate  transaminase | 0.004739373 | 18.02 | up |
| PP_3771[no_symbol] | hypothetical protein | 0.009296297 | 17.95 | up |
| PP_3214[no_symbol] | hypothetical protein | 0.027582581 | 17.76 | up |
| PP_2090[cobA-1] | uroporphyrin-III C-methyltransferase | 0.004635488 | 17.68 | up |
| PP_4241[no_symbol] | hypothetical protein | 0.007219487 | 17.63 | up |
| PP_1388[no_symbol] | EmrB/QacA family drug resistance transporter | 0.027002262 | 17.61 | up |
| PP_2685[no_symbol] | hypothetical protein | 0.028730815 | 17.58 | up |
| PP_3469[no_symbol] | hypothetical protein | 0.025881907 | 17.54 | up |
| PP_3325[no_symbol] | outer membrane ferric siderophore receptor | 0.049269983 | 17.50 | down |
| PP_4094[no_symbol] | hypothetical protein | 0.007897979 | 17.47 | up |
| PP_2612[no_symbol] | hypothetical protein | 0.007219487 | 16.98 | up |
| PP_1568[no_symbol] | hypothetical protein | 0.033019249 | 15.95 | down |
| PP_3213[no_symbol] | ABC transporter substrate-binding protein | 0.027537107 | 15.74 | up |
| PP_2708[no_symbol] | hypothetical protein | 0.035204703 | 15.70 | up |
| PP_0508[no_symbol] | hypothetical protein | 0.029942332 | 15.65 | down |
| PP_3301[no_symbol] | RND efflux membrane fusion protein | 0.027701588 | 15.61 | up |
| PP_3782[no_symbol] | hypothetical protein | 0.029679847 | 15.56 | down |
| PP_4093[no_symbol] | hypothetical protein | 0.01028539 | 15.38 | up |
| PP_0507[no_symbol] | ABC transporter ATP-binding protein | 0.031217936 | 14.80 | down |
| PP_0237[ssuA] | aliphatic sulfonate ABC transporter substrate-binding protein | 0.032261282 | 14.64 | down |
| PP_5360[no_symbol] | hypothetical protein | 0.046772989 | 14.11 | down |
| PP_0506[no_symbol] | hypothetical protein | 0.045713908 | 13.97 | down |
| PP_4239[no_symbol] | hypothetical protein | 0.027002262 | 13.90 | up |
| PP_4350[no_symbol] | aminotransferase | 0.032261282 | 13.75 | up |
| PP_3321[no_symbol] | hypothetical protein | 0.039058707 | 13.62 | down |
| PP_4408[no_symbol] | hypothetical protein | 0.044588509 | 13.52 | up |
| PP_0273[no_symbol] | hypothetical protein | 0.046895921 | 13.43 | down |
| PP_2847[ureJ] | HupE/UreJ protein | 0.011598709 | 13.40 | up |
| PP_2710[no_symbol] | hypothetical protein | 0.014977378 | 12.87 | up |
| PP_2988[no_symbol] | zinc-containing alcohol dehydrogenase | 0.022263901 | 12.84 | up |
| PP_1573[no_symbol] | major tail protein | 0.049269983 | 12.83 | down |
| PP_2709[no_symbol] | long-chain-fatty-acid--CoA ligase | 0.039373045 | 12.74 | up |
| PP_2845[ureC] | urease subunit alpha | 0.03752624 | 12.74 | up |
| PP_2846[ureE] | urease accessory protein UreE | 0.014977378 | 12.66 | up |
| PP_2652[no_symbol] | hydratase/decarboxylase | 0.029413097 | 12.46 | up |
| PP_2477[iorA] | isoquinoline 1-oxidoreductase subunit alpha | 0.017343809 | 12.05 | up |
| PP_4240[no_symbol] | microcin b17 processing protein mcbd | 0.020908959 | 11.80 | up |
| PP_3297[no_symbol] | hypothetical protein | 0.035204703 | 11.49 | up |
| PP_3212[no_symbol] | Rieske 2Fe-2S family protein | 0.025172775 | 11.32 | up |
| PP_3655[no_symbol] | cytosine/purines uracil thiamine allantoin  permease | 0.028530446 | 11.03 | up |
| PP_1930[arsR-1] | arsenic resistance transcriptional regulator | 0.039519052 | 10.36 | up |
| PP_4092[no_symbol] | ISPpu15. transposase Orf1 | 0.043013736 | 9.98 | up |
| PP_3947[nicA] | (2Fe-2S)-binding protein | 0.033851819 | 9.76 | up |
| PP_2711[no_symbol] | short chain dehydrogenase | 0.039224992 | 9.46 | up |
| PP_4351[no_symbol] | hypothetical protein | 0.043608716 | 9.44 | up |
| PP_5237[no_symbol] | hypothetical protein | 0.040362902 | 9.41 | up |
| PP_2625[no_symbol] | hypothetical protein | 0.043608716 | 9.41 | up |
| PP_4242[no_symbol] | hypothetical protein | 0.043608716 | 9.40 | up |
| PP_1254[xenA] | xenobiotic reductase A | 0.049596013 | 9.40 | up |
| PP_0782[no_symbol] | hypothetical protein | 0.043608716 | 9.01 | up |
| PP_3211[no_symbol] | ABC transporter ATP-binding protein | 0.049596013 | 8.56 | up |
